# Survival of western Gulf Coast Mottled Ducks (*Anas fulvigula*) in the path of a Category 4 hurricane

**DOI:** 10.1002/ece3.8276

**Published:** 2021-11-02

**Authors:** Kevin M. Ringelman, Elizabeth S. Bonczek, Joseph R. Marty, Ashley R. Booth, Alexandre L. Dopkin

**Affiliations:** ^1^ School of Renewable Natural Resources Louisiana State University AgCenter Baton Rouge Louisiana USA; ^2^ Louisiana Department of Wildlife and Fisheries Rockefeller Wildlife Refuge Grand Chenier Louisiana USA

**Keywords:** Gulf Coast, mortality, storm surge, tropical storm, waterfowl

## Abstract

Tropical cyclones are the most powerful storms on earth, causing catastrophic damage to human lives and infrastructure. Hurricanes also cause wildlife mortality when they make landfall, but the severity of these effects is difficult to quantify because data collection is either logistically impossible or deprioritized in the wake of human tragedy. On August 27, 2020, Hurricane Laura made landfall in southwestern Louisiana with maximum sustained winds of 241 kph (150 mph), making it one of the most powerful storms to strike the mainland United States. Hurricane Laura passed directly over the core breeding range of the western Gulf Coast population of Mottled Duck (*Anas fulvigula*), during a time when many adult birds were undergoing a simultaneous wing feather molt and were flightless. We used GPS‐GSM telemetry data to evaluate survival rates of adult female Mottled Ducks in late summer 2020 (bracketing August 27 by one month on either side) relative to the same period in 2018 and 2019. Mortality was lower in 2018 (12 out of 29; 41%) and 2019 (8 out of 28; 29%) than in 2020 (12 out of 18; 67%), and 7 out of 12 mortalities documented in 2020 occurred when Hurricane Laura made landfall. Survival analyses in program MARK confirmed lower survival probability in 2020, but there was overlap in 85% confidence intervals in all years. This single storm resulted in the death of ~40% of all marked birds in our sample, suggesting that hurricanes have the potential to influence population demographics. In addition, Hurricane Laura resulted in widespread habitat loss and degradation that has reduced available nesting habitat in 2021, and possibly for years to come. The acute and chronic effects of hurricanes may exacerbate Mottled Duck population declines, which may worsen in the face of increasingly frequent and more severe tropical storms.

## Graphical abstract

Mottled Ducks are endemic to the Gulf Coast of North America and a species of conservation concern in Louisiana. On August 27, 2020, Hurricane Laura made landfall in southwestern Louisiana as one of the most powerful hurricanes to strike the United States. Approximately ~40% of ducks marked with GPS transmitters perished in the storm, suggesting that tropical cyclones can have population‐level effects on demography.

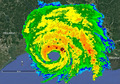

## 1 INTRODUCTION

Tropical cyclones are the most powerful storms on earth, capable of causing mass human mortality and widespread devastation of social and economic systems where they make landfall (Hossain & Mullick, 2020; Vigdor, 2008). Concerningly, global climate change and the resultant increase in sea surface temperatures have contributed to an increase in hurricane activity over the past half‐century (Saunders & Lea, 2008), and the frequency of powerful storms is expected to increase in the future (Knutson et al., 2013, 2015). While global attention has rightly focused on human suffering and economic devastation, hurricanes also have both acute and chronic impacts on local environmental conditions, including wildlife habitat destruction (Uriarte et al., 2019) and degradation of water quality (Hernández et al., 2020), which can percolate through the ecological trophic web for years (Stroud et al., 2019).

The impacts of hurricanes on wildlife are difficult to determine because: (a) detailed pre‐hurricane data often do not exist, (b) post‐hurricane data collection on wildlife is deprioritized in the wake of human tragedy, and (c) post‐hurricane data collection (e.g., on animal mortality) may be logistically impossible after a storm. In spite of these obstacles, Gunter and Eleuterius (1971) noted mass mortality (“thousands”) of eastern harvest mice (*Reithrodontomys humulis*) and dead raccoons (*Procyon lotor*) “every ten yards or so” following the passage of Hurricane Betsy along the Mississippi Coast in 1965. They also report the U.S. Army Corps of Engineers removing 28 tons of animals (primarily pets and livestock) following the 1969 landfall of Hurricane Camille in Mississippi, the second‐most powerful hurricane to strike the United States (Gunter & Eleuterius, 1971). Despite higher mobility than mammals, birds are also subject to local morality and displacement from hurricanes. Beach‐nesting and pelagic seabirds are commonly killed and/or blown inshore (Bugoni et al., 2007; Hall, 1981; Langham, 1986; Wiley & Wunderle, 1993). Even species further inland are at risk: Hooper et al. (1990) estimated that 63% of endangered Red‐cockaded Woodpeckers (*Picoides borealis*) in the path of Hurricane Hugo were killed in South Carolina in 1989. That being said, birds are highly mobile, and so most hurricane mortality events are modest in terms of population impact (i.e., a few hundred birds at mostBugoni et al., 2007; Cely, 1991; Ensminger & Nichols, 1957; Wiley & Wunderle, 1993). In addition to direct mortality, hurricanes can have long‐term impacts on bird populations through destruction and degradation of habitat required for nesting, roosting, and foraging (Wiley & Wunderle, 1993).

The Mottled Duck (*Anas fulvigula*) is a non‐migratory species of waterfowl endemic to the Gulf Coast of the United States with a range stretching from peninsular Florida in the east to the Laguna Madre region of Mexico in the west, with introduced populations in South Carolina and Georgia (Bonczek & Ringelman, 2021). The western Gulf Coast population of Mottled Ducks (hereafter “Mottled Ducks” for simplicity) extends through Alabama, and individuals rely primarily on coastal marsh habitats throughout the annual cycle (Baldassarre, 2014). Accordingly, Mottled Ducks are particularly susceptible to mortality and habitat loss caused by tropical storms and hurricanes. During the Atlantic hurricane season June 1–November 30, Mottled Ducks must nest (~35 days), raise a brood (~50 days), and also undergo a simultaneous wing feather molt during which they are flightless (~30 days) (Baldassarre, 2014). Moreover, because they spend their entire annual cycle in coastal marsh and associated habitats, chronic effects of habitat loss and degradation from hurricanes have the potential to affect Mottled Ducks for years after a storm passes. As year‐round residents of the Gulf Coast, Mottled Ducks should be well adapted to cope with hurricanes. Indeed, during the passage of Hurricane Audrey (Category 3) in southwest Louisiana in late June 1957, “a few mottle [sic] ducks…appeared to be making out very well in the water and on the drift” at Rockefeller Wildlife Refuge (hereafter “Rockefeller”) approximately 100 km from the center of the storm (Ensminger & Nichols, 1957). While the Mottled Ducks may have lost nests or broods during that June storm, nearly all adult female ducks would have been fully flighted (Stutzenbaker, 1988) and thus more capable of dealing with wind and storm surge.

In the pre‐dawn darkness of August 27, 2020, Hurricane Laura made landfall in southwestern Louisiana as a Category 4 storm with maximum sustained winds of 241 kph (150 mph), just short of Category 5 designation. As such, Hurricane Laura was tied for the strongest hurricane in recorded history to make landfall in Louisiana, and the 5th strongest hurricane to strike the continental United States. Hurricane Laura made landfall in Cameron, Louisiana (29°46′ N, 93°17′ W), passing directly over Rockefeller, and hurricane force winds extended 95 km from the center, affecting thousands of square kilometers of coastal marsh habitats. Unlike Hurricane Audrey in 1957, Laura struck in late summer, near the peak of the Mottled Duck flightless period, which typically spans August and September. Here, we used data collected from Mottled Ducks marked with GPS‐GSM transmitters in southwest Louisiana to evaluate survival during the passage of Hurricane Laura in late summer 2020, and compare those survival rates with the same two‐month period in 2018 and 2019.

## 2 METHODS

In August and September 2017–2019, we captured adult female Mottled Ducks in southwest Louisiana on Rockefeller Wildlife Refuge (29°42′ N, 92°52′ W) and nearby private property. We captured ducks by hand on moonless nights using spotlights and airboats; all ducks were in some stage of molt, and most were flightless. We fitted adult female Mottled Ducks with 21 g Ecotone GPS‐GSM Saker‐L and 18 g Crex transmitters attached in a Dwyer configuration (Dwyer, 1972) using Conrad–Jarvis 6.35‐mm black nylon automotive elastic with neoprene elastomer. We released birds on‐site that same evening after capture and marking. All protocols were approved by the Louisiana State University Institutional Animal Care and Use Committee (permit A2016‐27) and the Federal Bird Banding Lab (permit 06669). We programmed the GPS units to log locations every two hours during daylight, and locations were transmitted via the cellular network after every four fixes to an online data portal where birds were regularly monitored.

Here, we present survival analyses using telemetry data (2018–2020) collected from July 27 through September 27, which brackets the landfall of Hurricane Laura on August 27 by one month on either side. We included all available birds, including those that had been marked for more than one season. Most survival analyses based on telemetry data censor animals for which mortality cannot be confirmed (e.g., Varner et al., 2014) either through onboard mortality switches or by physically locating the dead animal. In our analyses, we counted birds that “disappeared” as mortalities during each of the three years under investigation; mortality date is thus associated with the last GPS‐GSM check‐in on record. Our reasoning is that a violent and acute weather event such as a Category 4 hurricane is more likely to result in bird mortality and subsequent preclusion from connecting with the cellular network (e.g., covered by debris, underwater) than bird survival concomitant with transmitter failure. This assumption is supported by the presence of surviving birds/transmitters that continued to communicate data through the cellular network after Hurricane Laura made landfall. We used the Nest Survival module in program MARK (White & Burnham, 1999) accessed through the RMark package (Laake, 2013) in program R (R Core Team, 2018) to evaluate survival rates during the late summer period in question. We opted to use this survival modeling framework because it permits staggered entry (ducks were marked at different times) and, unlike known‐fate models, does not require that animals are monitored in discrete intervals (we had variable check‐in periods because of duck movements and cell service coverage) (Rotella, 2021). The Nest Survival module in MARK requires three dates for each individual: (a) “FirstFound,” which we fixed to July 27, the beginning of the temporal window; (b) “LastPresent,” which was the date of last GPS‐GSM check‐in for mortalities, and September 27 for survivors; (c) “LastChecked,” which was the date after the last check‐in for mortalities, and September 27 for survivors. We evaluated annual differences in Mottled Duck survival; 2020 was the only year a hurricane struck southwest Louisiana. In addition, as a way to specifically diagnose the magnitude of the effect that hurricane Laura had on Mottled Duck survival, we created a binary dummy variable to delineate whether a duck experienced a hurricane (defined as 2 days before and after landfall) and included this in our set of survival models. While we clearly anticipated this to be the best‐fitting survival model in our candidate set, comparing it using evidence ratios to models without the hurricane dummy variable provided a useful way to judge the magnitude of difference in survival rates caused by the hurricane. We ranked models using AICc scores (Burnham & Anderson, 2002) and present 85% confidence intervals that are more appropriate than 95% confidence intervals when using an AIC‐based approach to model selection (Arnold, 2010).

To quantitatively infer the biological impacts of Hurricane Laura as it made landfall, we also have water‐level data collected at Rockefeller (within 20 km of most coastal Mottled Duck locations in representative coastal marsh habitat) for the period between July 27 and September 27, 2020. A Rugged Troll 100 water‐level logger (In‐Situ Inc.) was deployed (29°37′ N, 92°40′ W) in a PVC well installed to a depth of one meter below the soil surface (as in Folse et al., 2008). Height of water above the logger and water temperature were recorded every 30 min. Water‐level data were corrected in post‐processing with Win‐Situ 5 software (In‐Situ 2012) using concurrent barometric pressure readings from a BaroTroll 100 barometric pressure logger (In‐Situ Inc.) deployed within 3.4 km of the water‐level logger. Following post‐processing with barometric pressure readings, data were corrected for depth below the soil surface to convert height of water above the logger to height of water above the soil surface.

## 3 RESULTS

**FIGURE 1:**
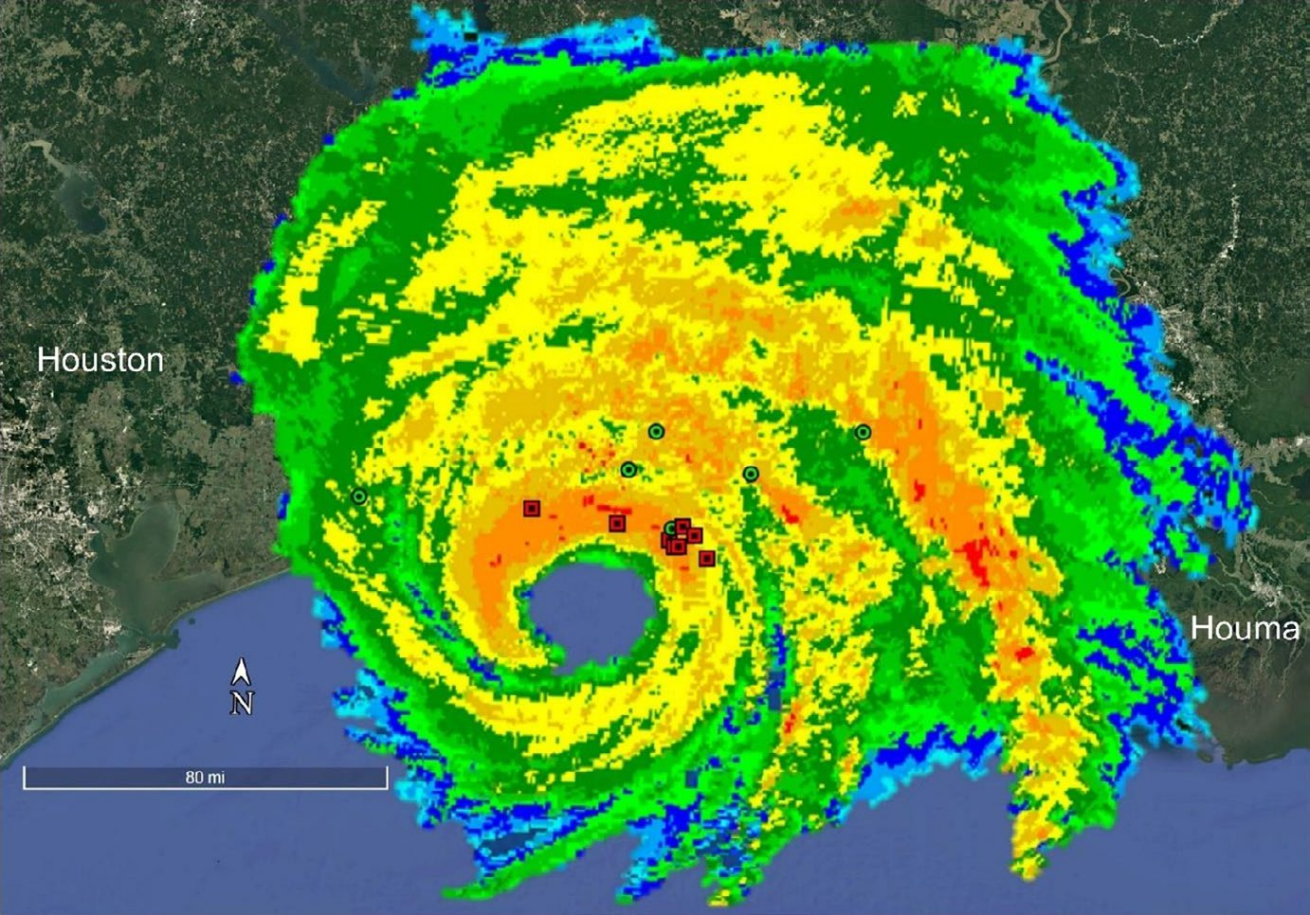
Locations of Mottled Ducks (*Anas fulvigula*) marked with GPS‐GSM transmitters in Louisiana and Texas, USA, superimposed on a composite radar image (data via National Oceanic and Atmospheric Administration) of Hurricane Laura as it made landfall on August 27, 2020. Red squares indicate mortalities, and green circles indicate birds that survived

**FIGURE 2:**
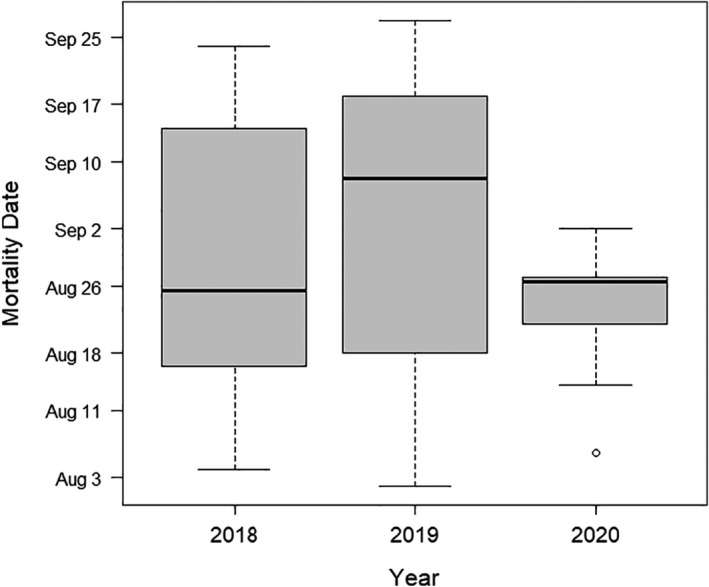
Temporal distribution of observed Mottled Duck (*Anas fulvigula*) mortalities in August and September 2018–2020. Data were collected from ducks marked with GPS‐GSM transmitters in Louisiana and Texas, USA (2018: *n* = 29, 2019: *n* = 28, 2020: *n* = 18)

Our sample size consisted of 29, 28, and 18 Mottled Ducks alive on July 27 in 2018, 2019, and 2020, respectively. Eight out of the 13 birds that checked in immediately prior to the storm were located within 10 km of the coast at the time of landfall (Figure 1). Three were located ~25–40 km inland in areas near the path of the hurricane (e.g., Cameron Prairie National Wildlife Refuge in Bell City, LA, and south of Bell City, LA), and 2 ducks were located ~50–125 km away from the path (e.g., JD Murphree Wildlife Management Area in Port Arthur, TX, White Lake Wetlands Conservation Area in Gueydan, LA). Over the course of all three years, the observed “mortality” (which includes disappearances) was high (43%) between July 27 and September 27, which was expected given that most ducks likely underwent flightless wing molt during this period and were exposed to numerous predators, especially American alligators (*Alligator mississippiensis*). Observed proportional mortality was lower in 2018 (41%) and 2019 (29%) than in 2020 (67%) (Table 1). Survival analysis in MARK confirmed a lower probability of survival in 2020, but there was overlap in 85% confidence intervals among all years (Table 1). Mortality (last GPS‐GSM check‐in) was distributed throughout the temporal window in 2018 and 2019, but was concentrated around August 26 in 2020 (Figure 2), with 7 out of 12 mortalities on August 25 or 26. The survival model that included the dummy variable for hurricane presence provided the best fit to the data (Table 2), and was ~12 times more likely than the model containing year and ~32 times more likely than the null model indicating that the presence of a hurricane was a powerful predictor of Mottled Duck survival.

**TABLE 1:** Raw survival data for Mottled Ducks, and results from program MARK parsing survival rates by year

Year	Number of Mottled Ducks alive at start	Number of mortalities	Estimated probability of surviving sample period	85% CI
2018	29	12	0.110	0.029–0.334
2019	28	8	0.201	0.063–0.483
2020	18	12	0.010	0.001–0.121

**TABLE 2:** Candidate models describing survival rates of Mottled Ducks as a function of year and the presence of a hurricane

Model	Degrees of freedom	AICc	ΔAICc	AICc weight
Hurricane	2	372.1	0.0	0.897
Year	3	377.0	4.9	0.075
Null	1	379.0	6.9	0.028

**FIGURE 3:**
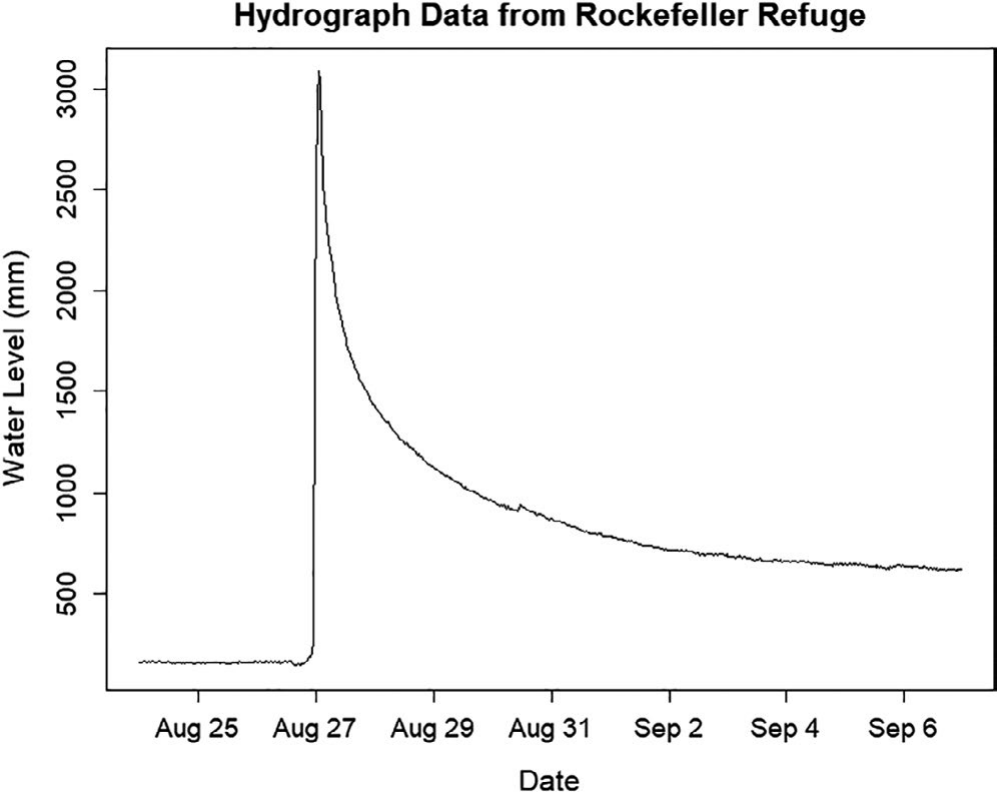
Hydrograph data collected from Rockefeller Wildlife Refuge, Louisiana, USA, as Hurricane Laura made landfall on August 27, 2020

Hydrograph data collected on‐site at Rockefeller indicated water levels began to slowly increase midday on August 26, 2020, with storm surge striking close to midnight on the morning of August 27 (Figure 3). Water levels increased 2.8 m between the hours of 0415 UTC on August 26 and 0815 UTC on August 27, with storm surge peaking at levels ~20 times higher than baseline (3.1 m in comparison with ~0.2 m), and remaining ~4 times higher for weeks afterward. Similarly, a United States Geological Survey water‐level sensor at Rockefeller (29°42′ N, 92°46′ W) showed a peak inundation of 2.8 m (Pasch et al., 2021). The National Oceanic and Atmospheric Administration estimated storm surge as high as 5.5 m near landfall in Cameron, with surge of 1–2 m impacting the entirety of coastal Louisiana and much of the southeast Texas coast (Pasch et al., 2021). In major inundation zones, only 2 out of 10 birds were known to survive (Figure 1).

## 4 DISCUSSION

The passage of Hurricane Laura appears to have caused ~40% mortality of marked female Mottled Ducks in southwest Louisiana in the days surrounding August 27, 2020. Flighted Mottled Ducks tend to make limited movements and have small home ranges of only a few hectares throughout the annual cycle (Bonczek et al., unpublished). Thus, we were unable to definitively determine which individuals were molting and unable to escape the storm, versus those that chose to ride it out (*sensu* Ensminger & Nichols, 1957). Based on movement data before and after the storm, we believe that at least 2, and as many as 5 birds were flighted, of which 3 survived. We documented no long‐distance movements to escape the path of the hurricane; however, during the course of the storm two individuals each moved 4 km inland, but it is unclear whether this was a deliberate movement or associated with storm surge. Mottled Ducks are particularly susceptible to hurricanes during molt not only because they are flightless and unable to move out of the path of storms, but they also actively leave inland habitats in favor of those on the coast to molt (Bonczek, unpublished data; Moon et al., 2015) and have a high degree of molt site fidelity (Wehland, 2012). Indeed, 4 out of 29, 8 out of 28, and 5 out of 18 Mottled Ducks made movements of ~25–110 km in August 2018, 2019, and 2020, respectively in order to access coastal habitat.

We acknowledge that survival rates and behavior of ducks equipped with telemetry transmitters may not be representative of unmarked birds (Kesler et al., 2014), but because we used the same attachment methods each season, between‐year comparisons should be valid. Still, two possibilities for transmitter effects remain: (a) It is possible that marked birds (or transmitters) were suffering from nonlethal deleterious effects that predisposed them to mortality when Hurricane Laura hit, or conversely (b) given that all of the birds (and transmitters) facing Hurricane Laura had already survived nearly a full year or more, they may actually represent a subset of particularly high‐quality individuals. We are not able to parse these potential transmitter effects, but we acknowledge that either is possible. Regardless, if our telemetry data were even moderately representative of the larger unmarked population, our results imply that substantial numbers of Mottled Ducks in the affected region may have perished in the storm. This is of particular concern because the Mottled Duck population in Louisiana has declined to less than half of what it was a decade ago (Bonczek & Ringelman, 2021), and Hurricane Laura passed directly over the core of their breeding range. This emphasizes the potential detrimental effect of severe, stochastic, point‐source mortality events on at‐risk wildlife populations (Hooper et al., 1990), although quantitative evidence for such remains scant.

The habitat in southwest Louisiana was devastated by Hurricane Laura's record storm surge, which scoured the landscape and inundated freshwater and upland areas for weeks or months. Previous research has indicated that hurricanes can fundamentally alter estuarine habitats and food webs, with trickle‐up trophic effects on waterfowl populations (Stroud et al., 2019). In affected areas of southwest Louisiana, vegetation that is potentially used by Mottled Ducks for nesting (Bonczek & Ringelman, 2021) was only beginning to rebound in May 2021, and some habitats (e.g., constructed earthen terraces) were functionally unavailable (Dopkin et al., unpublished). Within Rockefeller, impacts of salt stress from storm surge and prolonged flooding resulted in widespread plant death leading to peat collapse (i.e., Chambers et al., 2019) at several coastal marsh sites (Booth et al., unpublished). In ecosystems with limited sediment inputs like those at Rockefeller, peat collapse and subsequent erosion are directly linked to marsh fragmentation (DeLaune et al., 1994) and permanent wetland loss (e.g., Cahoon et al., 2003), both of which are detrimental to breeding and wintering waterfowl. An unforeseen knock‐on consequence of habitat loss and degradation in the region is displacement of cattle ranching operations, which faced extreme economic pressure to heavily graze any suitable grasslands that remained (Dopkin et al., unpublished). Thus, the negative impacts of Hurricane Laura on Mottled Ducks in southwest Louisiana were both direct and indirect, as well as acute and chronic. Such powerful storms are forecast to increase in both frequency and intensity in the future (Knutson et al., 2013, 2015). These storms, whose effects are intensified by regionally high rates of sea‐level rise (Kolker et al., 2011) and declines in marsh resilience (Stagg et al., 2020), pose a significant threat to the coastal marsh systems on which Mottled Duck populations rely. Our analyses provide some of the first quantitative data on how hurricanes affect avian survival, and we encourage the establishment of monitoring programs (e.g., https://gomamn.org/) to provide similar data in the future.

## CONFLICT OF INTEREST

The authors have no conflict of interest to declare.

## AUTHOR CONTRIBUTIONS

**Kevin M. Ringelman:** Conceptualization (lead); data curation (equal); formal analysis (equal); funding acquisition (lead); methodology (equal); project administration (lead); supervision (lead); visualization (equal); writing‐original draft (lead). **Elizabeth S. Bonczek:** Conceptualization (supporting); data curation (equal); formal analysis (equal); funding acquisition (supporting); investigation (lead); methodology (equal); writing‐review & editing (equal). **Joseph R. Marty:** Investigation (supporting); resources (lead); supervision (supporting); visualization (equal); writing‐review & editing (equal). **Ashley R. Booth:** Data curation (supporting); investigation (supporting); writing‐review & editing (equal). **Alexandre L. Dopkin:** Visualization (equal); writing‐review & editing (equal).

## Data Availability

All data are available via Dryad at https://doi.org/10.5061/dryad.0zpc866z7.

## References

[ece38276-bib-0001] Arnold, T. W. (2010). Uninformative parameters and model selection using Akaike's information criterion. Journal of Wildlife Management, 74, 1175–1178. 10.1111/j.1937-2817.2010.tb01236.x

[ece38276-bib-0002] Baldassarre, G. (2014). Ducks, geese, and swans of North America. A wildlife management institute book. Johns Hopkins University Press.

[ece38276-bib-0003] Bonczek, E. S. , & Ringelman, K. M. (2021). Breeding ecology of mottled ducks: A review. The Journal of Wildlife Management, 85(5), 825–837. 10.1002/jwmg.22048

[ece38276-bib-0004] Bugoni, L. , Sander, M. , & Costa, E. S. (2007). Effects of the first southern Atlantic hurricane on Atlantic petrels (*Pterodroma incerta*). The Wilson Journal of Ornithology, 119, 725–729. 10.1676/06-141.1

[ece38276-bib-0005] Burnham, K. P. , & Anderson, D. R. (2002). Model selection and multimodel inference: A practical information‐theoretic approach. Springer‐Verlag.

[ece38276-bib-0006] Cahoon, D. R. , Hensel, P. , Rybczyk, J. , McKee, K. L. , Proffitt, C. E. , & Perez, B. C. (2003). Mass tree mortality leads to mangrove peat collapse at Bay Islands, Honduras after Hurricane Mitch. Journal of Ecology, 91, 1093–1105. 10.1046/j.1365-2745.2003.00841.x

[ece38276-bib-0007] Cely, J. E. (1991). Wildlife effects of hurricane Hugo. Journal of Coastal Research, Special Issue (8), 319–326.

[ece38276-bib-0008] Chambers, L. G. , Steinmuller, H. E. , & Breithaupt, J. L. (2019). Toward a mechanistic understanding of “peat collapse” and its potential contribution to coastal wetland loss. Ecology, 100, e02720. 10.1002/ecy.2720 30933312 PMC6850666

[ece38276-bib-0009] DeLaune, R. D. , Nyman, J. A. , & Patrick, W. H. Jr (1994). Peat collapse, ponding, and wetland loss in a rapidly submerging coastal marsh. Journal of Coastal Research, 10, 1021–1030.

[ece38276-bib-0010] Dwyer, T. J. (1972). An adjustable radio‐package for ducks. Bird‐Banding, 43, 282–284. 10.2307/4511905

[ece38276-bib-0011] Ensminger, A. B. , & Nichols, L. G. (1957). Hurricane damage to Rockefeller Refuge. In Proceedings of the annual conference of the Southeastern Association of Game and Fish Commission (Vol. 11, pp. 52–56).

[ece38276-bib-0012] Folse, T. M. , West, J. L. , Hymel, M. K. , Troutman, J. P. , Sharp, L. A. , Weifenbach, D. K. , McGinnis, T. E. , Rodrigue, L. B. , Boshart, W. M. , Richardi, D. C. , Miller, C. M. , & Wood, W. B. (2008, revised 2012). Wetlands: Methods for site establishment, data collection, and quality assurance/quality control. In A standard operating procedures manual for the Coast‐wide Reference Monitoring System (207 pp). Louisiana Coastal Protection and Restoration Authority.

[ece38276-bib-0013] Gunter, G. , & Eleuterius, L. N. (1971). Some effects of hurricanes on the terrestrial biota, with special reference to Camille. Gulf and Caribbean Research, 3, 283–289.

[ece38276-bib-0014] Hall, G. A. (1981). The changing seasons. American Birds, 35, 150–156.

[ece38276-bib-0015] Hernández, W. J. , Ortiz‐Rosa, S. , Armstrong, R. A. , Geiger, E. F. , Eakin, C. M. , & Warner, R. A. (2020). Quantifying the effects of Hurricanes Irma and María on coastal water quality in Puerto Rico using moderate resolution satellite sensors. Remote Sensing, 12, 964.

[ece38276-bib-0016] Hooper, R. G. , Watson, J. C. , & Escano, R. E. (1990). Hurricane Hugo's initial effects on red‐cockaded woodpeckers in the Francis Marion National Forest. In Transactions of the North American wildlife and natural resources conference (Vol. 55, pp. 220–224).

[ece38276-bib-0017] Hossain, I. , & Mullick, A. R. (2020). Cyclone and Bangladesh: A historical and environmental overview from 1582 to 2020. International Medical Journal, 25, 2595–2614.

[ece38276-bib-0018] Kesler, D. C. , Raedeke, A. H. , Foggia, J. R. , Beatty, W. S. , Webb, E. B. , Humburg, D. D. , & Naylor, L. W. (2014). Effects of satellite transmitters on captive and wild Mallards. Wildlife Society Bulletin, 38, 557–565. 10.1002/wsb.437

[ece38276-bib-0019] Knutson, T. R. , Sirutis, J. J. , Vecchi, G. A. , Garner, S. , Zhao, M. , Kim, H.‐S. , Bender, M. , Tuleya, R. E. , Held, I. M. , & Villarini, G. (2013). Dynamical downscaling projections of twenty‐first‐century Atlantic hurricane activity: CMIP3 and CMIP5 model‐based scenarios. Journal of Climate, 26, 6591–6617. 10.1175/JCLI-D-12-00539.1

[ece38276-bib-0020] Knutson, T. R. , Sirutis, J. J. , Zhao, M. , Tuleya, R. E. , Bender, M. , Vecchi, G. A. , Villarini, G. , & Chavas, D. (2015). Global projections of intense tropical cyclone activity for the late twenty‐first century from dynamical downscaling of CMIP5/RCP4. 5 scenarios. Journal of Climate, 28, 7203–7224. 10.1175/JCLI-D-15-0129.1

[ece38276-bib-0021] Kolker, A. S. , Allison, M. A. , & Hameed, S. (2011). An evaluation of subsidence rates and sea‐level variability in the northern Gulf of Mexico. Geophysical Research Letters, 38, L21404. 10.1029/2011GL049458

[ece38276-bib-0022] Laake, J. L. (2013). RMark: An R Interface for analysis of capture‐recapture data with MARK. Alaska Fisheries Science Center Report 2013‐01. NOAA, National Marine Fisheries Service.

[ece38276-bib-0023] Langham, N. (1986). The effect of cyclone ‘Simon’ on terns nesting on One Tree Island, Great Barrier Reef, Australia. Emu‐Austral Ornithology, 86, 53–57. 10.1071/MU9860053

[ece38276-bib-0036] Moon, J. A. , Haukos, D. A. , & Conway, W. C. (2015). Mottled duck (*Anas fulvigula*) movements in the Texas Chenier Plain region. Journal of the Southeastern Association of Fish and Wildlife Agencies, 2, 255–261.

[ece38276-bib-0024] Pasch, R. J. , Berg, R. , Roberts, D. P. , & Papin, P. P. (2021). National Hurricane Center tropical cyclone report: Hurricane Laura (AL13202020) 20‐29 August 2020 (75 pp).

[ece38276-bib-0025] R Core Team (2018). R: A language and environment for statistical computing. R Foundation for Statistical Computing. https://www.R‐project.org/

[ece38276-bib-0026] Rotella, J. (2021). Nest survival models. In E. G. Cooch & G. C. White (Eds.), Program MARK. A gentle introduction. https://www.phidot.org/software/mark/docs/book/

[ece38276-bib-0027] Saunders, M. A. , & Lea, A. S. (2008). Large contribution of sea surface warming to recent increase in Atlantic hurricane activity. Nature, 451, 557–560. 10.1038/nature06422 18235498

[ece38276-bib-0028] Stagg, C. L. , Osland, M. J. , Moon, J. A. , Hall, C. T. , Feher, L. C. , Jones, W. R. , Couvillion, B. R. , Hartley, S. B. , & Vervaeke, W. C. (2020). Quantifying hydrologic controls on local‐ and landscape‐scale indicators of coastal wetland loss. Annals of Botany, 125, 365–376. 31532484 10.1093/aob/mcz144 PMC7442328

[ece38276-bib-0029] Stroud, C. M. , Caputo, C. E. , Poirrier, M. A. , Reynolds, L. A. , & Ringelman, K. M. (2019). Bluebills and bayou bivalves: Hurricane‐driven trophic cascades affect wintering abundance of Lesser Scaup in Louisiana. Ecosphere, 10, e02829. 10.1002/ecs2.2829

[ece38276-bib-0030] Stutzenbaker, C. D. (1988). The mottled duck, its life history, ecology and management. Texas Parks and Wildlife Department.

[ece38276-bib-0031] Uriarte, M. , Thompson, J. , & Zimmerman, J. K. (2019). Hurricane María tripled stem breaks and doubled tree mortality relative to other major storms. Nature Communications, 10, 1–7. 10.1038/s41467-019-09319-2 PMC6433954 30911008

[ece38276-bib-0032] Varner, D. M. , Hepp, G. R. , & Bielefel, R. R. (2014). Annual and seasonal survival of adult female Mottled Ducks in southern Florida, USA. Condor, 116, 134–143. 10.1650/CONDOR-13-078.1

[ece38276-bib-0033] Vigdor, J. (2008). The economic aftermath of Hurricane Katrina. Journal of Economic Perspectives, 22, 135–154. 10.1257/jep.22.4.135

[ece38276-bib-0037] Wehland, E. M. (2012). Survival and post‐breeding habitat use of mottled ducks in the western Gulf Coast. In Dissertation. Texas A&M University.

[ece38276-bib-0034] White, G. C. , & Burnham, K. P. (1999). Program MARK: Survival estimation from populations of marked animals. Bird Study, 46, 120–139. 10.1080/00063659909477239

[ece38276-bib-0035] Wiley, J. W. , & Wunderle, J. M. (1993). The effects of hurricanes on birds, with special reference to Caribbean islands. Bird Conservation International, 3, 319–349. 10.1017/S0959270900002598

